# Digital basierte klinisch-orientierte Anatomie: Zukunft der Lehre

**DOI:** 10.1007/s00104-024-02211-w

**Published:** 2025-01-08

**Authors:** Esther C. Maier, Veysel Ödemis, Anja U. Bräuer

**Affiliations:** Abteilung für Anatomie, Fakultät VI Medizin und Gesundheitswissenschaften, Carl von Ossietzky Universität Oldenburg, Carl-von-Ossietzky-Str 9–11, 26129 Oldenburg, Deutschland

**Keywords:** Anatomischer Unterricht, Studierendenerwartungen, Lernmodalität, Digitale Lehre, Mixed Reality, Anatomical teaching, Student expectations, Learning modality, Digital teaching, Mixed reality

## Abstract

Die universitäre Lehre befindet sich im Umbruch. Steigende Studierendenzahlen sowie die voranschreitende Digitalisierung des Alltags führen auch in der Lehre zur Erprobung verschiedenster neuer Lehr- und Lernformate. Dieser Artikel gibt eine Übersicht über Hintergründe und Ansätze, die genutzt werden, um anatomischen Unterricht unter Einsatz digitaler Lernmethoden effektiv und effizient zu gestalten und den Erwartungen der Studierenden gerecht zu werden.

## Abstract (en)

University teaching is undergoing radical changes. Rising student numbers and the progressive digitalization of routine daily life are also leading to the testing of various new teaching and learning formats. This article provides an overview of the reasons for and approaches used to effectively and efficiently organize teaching of anatomy using digital learning methods and to fulfil the expectations of students.

In den letzten Jahren ist die Digitalisierung in vielen Lebensbereichen massiv vorangeschritten. In der universitären Lehre hielten während der SARS-CoV 2(„severe acute respiratory syndrome coronavirus type 2“)-Pandemie vermehrt digitale Lehr- und Lernmethoden Einzug, so auch in der Anatomie. Ein Teil dieser neuen oder weiterentwickelten digitalen Formate hat auch weiterhin seinen Platz im anatomischen Unterricht. Bei all den neu entwickelten Lern- und Lehransätzen stellt sich natürlich die Frage, ob überhaupt die Notwendigkeit besteht, anatomische Lehre neu zu gestalten.

## Anatomische Lehre: Warum nicht einfach weiter so?

Bei Erwähnung des Studiums der Humanmedizin werden bei den meisten Menschen Assoziationen an den Präparierkurs und die damit verbundene Dissektion von Spenderkörpern wach. Dies verdeutlicht, wie stark die Anatomie die Ausbildung von Mediziner:innen prägt. Fundierte Kenntnisse der Anatomie sind gerade in operativen Fächern unerlässlich und auch für die Studierenden von entscheidender Bedeutung. Dennoch ist die Anatomie nur eines von vielen wichtigen vorklinischen Fächern. Das relevante Kernwissen hat sowohl in den Naturwissenschaften als auch in den klinischen Fächern enorm zugenommen, während die Lehrstunden im Fach Anatomie in den letzten hundert Jahren kontinuierlich zurückgegangen sind. So wurden um 1930 durchschnittlich noch über 800 h Anatomie unterrichtet, mittlerweile nur noch etwa 200 h [1].

Eine Reduktion des anatomischen Unterrichts muss nicht zwangsläufig eine Verschlechterung bedeuten, dennoch nehmen die Klagen über ärztliche Kunstfehler zu [2, 3]. Kliniker:innen berichten vermehrt von ungenügenden oder mangelhaften Anatomiekenntnissen der Studierenden [4–7]. Auch die Studierenden selbst oder Ärzt:innen in der frühen Phase der Facharztausbildung berichten, dass sie ihr Vorwissen als ungenügend empfinden [8, 9].

Insgesamt legt dies nahe, dass die Reduktion der anatomischen Lehrinhalte dazu führt, dass angehende Mediziner:innen nicht genügend anatomisches Wissen erworben haben und somit nicht optimal auf die berufliche Praxis vorbereitet sind. Fehlende anatomische Kenntnisse gefährden daher die Patient:innengesundheit. Medizinstudierende haben im Studium eine hohe Arbeits- und Prüfungsbelastung und das eng getaktete Kurrikulum lässt einen Ausbau der anatomischen Lehrstunden nicht zu. Folglich ist es umso wichtiger, ein Kernkurrikulum mit dazu passenden Lehrmethoden zu entwickeln, die in der verkürzten Zeit die Inhalte effektiv und effizient vermitteln und die Studierenden mit den nötigen anatomischen Kompetenzen in den Klinikteil der Ausbildung entlässt. Es ist daher nicht verwunderlich, dass an den anatomischen Instituten weltweit ein großes Interesse daran besteht, herauszufinden, welche Methoden geeignet sind, die knapp bemessene Zeit optimal zur Wissensvermittlung zu nutzen.

## Die Studierenden: ihre Erwartungen an Lehre

Nicht nur die zur Verfügung stehende Zeit für die Vermittlung von Inhalten hat sich verändert, sondern auch die Studierenden, ihre Lerngewohnheiten und Erwartungen an die Lehre haben sich gewandelt [10, 11]. In der heutigen Zeit bevorzugen Studierende flexible Lernumgebungen und erwarten den Einsatz moderner IT-Technologien zur Unterstützung des Unterrichts [12, 13]. Man könnte jetzt sagen „So what!“, allerdings zeigen Studien, dass die Motivation der Studierenden entscheidend Einfluss auf ihren Lernerfolg hat [10, 14, 15].

Daher sollte moderne Lehre auf die Lernpräferenzen der Studierenden eingehen und durch den Einsatz verschiedenster Lehrmethoden gewährleisten, dass sich unterschiedliche Lerntypen wiederfinden. Darüber hinaus werden Kompetenzen für den Berufsalltag vermittelt, etwas, dass die Anatomie mit ihrem auch praktischen Fokus schon immer geleistet hat.

## Anatomische Lehre: „best practice“

In Deutschland wird derzeit die ärztliche Approbationsordnung neu gefasst [16]. Das umstrukturierte Kurrikulum sieht eine engere Verzahnung von vorklinischen mit klinischen Fächern vor, wie es heute schon in vielen Modellstudiengängen praktiziert wird.

In der anatomischen Lehrforschung liegt seit einiger Zeit der Schwerpunkt vermehrt auf zwei Themen: zum einen, wie sich der Einsatz unterschiedlicher Lernmodalitäten auf den Lernerfolg der Studierenden auswirkt, und zum anderen, wie anatomische Lehre in die Kurrikula integriert und longitudinal mit klinischen Fächern verzahnt werden kann. Befragt man Lehrende ist der recht einstimmige Konsensus, dass der Goldstandard der anatomischen Lehre die Dissektion ist. Allerdings zeigen wissenschaftliche Untersuchungen nur gering bessere oder gleichwertige Lerneffekte der Dissektion im Vergleich zu Prosektion oder digitalen Lerntools [17], was aber auch daran liegen mag, dass meist die kurzfristige Wissenswiedergabe überprüft wird [18].

Digitale Lehrmethoden können klassische Lehrmethoden ergänzen, aber nicht ersetzen

Ob die Dissektion langfristig zu einem besseren topographisch-anatomischen Verständnis führt, ist nicht hinreichend erforscht. Allerdings gab es um die Jahrtausendwende vermehrt Bestrebungen, Anatomie ohne Körperspende zu lehren, eine ökonomische Sparmaßnahme, die jetzt teils wieder rückgängig gemacht wird [19, 20]. Dies ist eine Reaktion auf vermehrte Klagen der Kliniker über schlechtere Anatomiekenntnisse der Studierenden seit Fehlen der Dissektion, zum anderen wird auch den Studierenden Rechnung getragen, die sich einen Präparierkurs wünschen [21, 22]. Dies stützt die Rolle der Dissektion als zentrales Element der anatomischen Ausbildung.

Für die meisten Studierenden ist der Unterricht der Anatomie, sei es mittels Dissektion oder Prosektion, auch der erste Kontakt mit dem Tod. Darüber hinaus sammeln sie im Rahmen der Pro- oder Dissektion erste Erfahrungen mit medizinethischen Fragen. Des Weiteren zeigen Studien, dass kognitive Fähigkeiten und manuelle Geschicklichkeit bei der Dissektion erworben werden [20, 23, 24]. Die anatomische Lehre trägt so auch zur berufsständischen Entwicklung der Studierenden bei.

Konsensus in der anatomischen Lehrforschung ist aktuell, dass ein multimodaler Ansatz am besten geeignet ist, die vielfältigen Inhalte der Anatomie zu vermitteln [25, 26]. Bereits in der vorklinischen Phase soll hier ein Praxisbezug hergestellt werden.

## Digitale anatomische Lehre: Ein Zukunftsmodell?

**Abb. 1:**
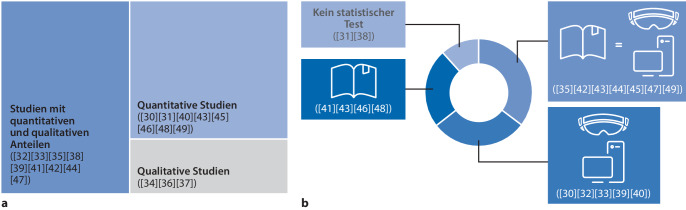
Übersicht und Hauptmerkmale von Studien zu Augmented Reality (AR) und Virtual Reality (VR) in der anatomischen Lehre [30–49]. **a** Übersicht über Studien, **b** Übersicht über quantitative Studienergebnisse

In den vergangenen zwanzig Jahren wurden vermehrt digitale Methoden in der anatomischen Lehre getestet und eingesetzt. Diese bieten innovative und interaktive Lernerfahrungen, die es den Studierenden ermöglichen, sich mit virtuellen anatomischen Modellen und medizinischen Szenarien auseinanderzusetzen (Abb. 1; [27–29]). Die Grundidee dahinter ist so einfach wie überzeugend: Dinge lassen sich virtuell dreidimensional besser darstellen als auf Papier. Zudem kann mehrmals etwas ausprobiert werden, ohne die Struktur unwiederbringlich zu schädigen.

Besonders großes Interesse besteht an der Nutzung von Methoden und Techniken, in denen in erweiterter (Mixed Reality, MR) oder virtueller Realität (Virtual Reality, VR) gelernt wird [50].

**Abb. 2:**
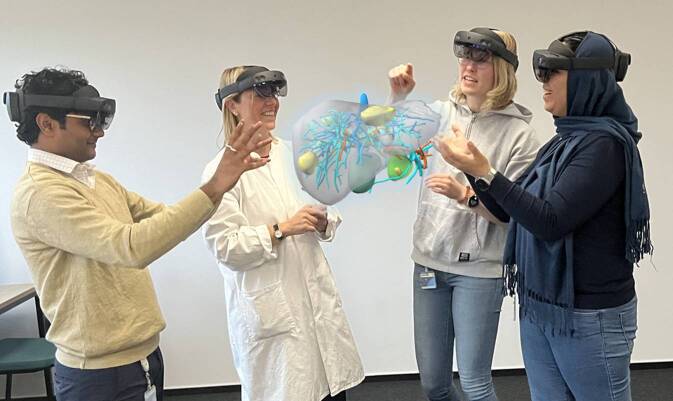
Unterrichtseinheit zur Leberanatomie mit AR(Augmented-Reality)-Brillen; die Lehrende und die Studierenden können gemeinsam an einem patientenspezifischen 3‑D-Modell Besonderheiten des Falles diskutieren

In der anatomischen Lehre wird ein breites Spektrum an MR-Visualisierungstechnologien eingesetzt. Viele davon funktionieren auf 2‑D-Geräten wie Smartphones, Tablets oder PCs und bieten ein wenig immersives Erlebnis. Andere hingegen nutzen Head-Mounted Displays (HDMs; Abb. 2), wo sowohl die realen als auch die digitalen Objekte in der gleichen Umgebung manipuliert werden können [27–29, 42, 51, 52]. Studien (Abb. 1), die digitale und AR/VR-Tools für das anatomische Lernen untersuchen, berichten durchweg über eine hohe Akzeptanz der neuen Lernumgebungen bei den Studierenden. Die Studierenden entwickeln ein besseres Verständnis der anatomischen Strukturen in drei Dimensionen [44, 47] und verbessern so ihr topographisch-anatomisches Verständnis. Der Effekt auf Wissenszuwachs und Lerneffektivität variiert stark. Der Wissensgewinn wird meist in einem Test direkt nach der Lerneinheit überprüft, wobei teils signifikante Unterschiede auftreten. Einige Studien zeigen, dass digitale Formate effektiver sind als traditionelle Methoden, während andere keinen Unterschied zwischen den beiden Ansätzen feststellen können. Ein Teil der Studien zeigt keinen Unterschied in den Testergebnissen der Studierenden zwischen den Methoden (Abb. 1). Allerdings ist die Auswahl der Wissensfragen oft begrenzt oder in ihrer Gewichtung so, dass eine Lernmethode besser zur Abfrage passt [41]. Wenn es ein „constructive alignment“ zwischen Lehr- und Prüfungsform gibt, schneiden die Studierenden in dieser Gruppe im Posttest besser ab. Fähigkeiten wie kommunikative und praktisch-handwerkliche Kompetenzen werden nicht quantitativ erfasst und fließen daher nicht in die Bewertung ein. Die qualitative Evaluierung konzentriert sich auf die fachliche Methodik sowie die Zufriedenheit und Motivation der Studierenden. Es zeigt sich, dass neu entwickelte digitale Lerntools die Motivation, den Spaß und das Engagement der Studierenden steigern. Dabei ist zu beachten, dass die Teilnahme meist freiwillig ist und oft Studierende höherer Semester partizipieren. Neue Lernmethoden sind oft spannender als bekannte Formate, was bedeutet, dass in longitudinalen Kurrikula die eingesetzten Lernmodalitäten im ersten Studienjahr anders gestaltet sein sollten als in späteren Jahren. Technische Innovationen können in höheren Semestern zusätzliche Impulse geben und das Engagement der Studierenden fördern.

Ein Nachteil von HMDs sind Schwindel und Kopfschmerzen („cybersickness“, CS), die bei VR oder bildreicher MR häufiger auftreten als bei Standard-MR [53]. Dies sollte bei der Konzeption der Lehreinheit berücksichtigt werden, indem eine Überladung mit virtuellen Bildern vermieden und die Dauer angemessen gestaltet wird. MR könnte gegenüber reiner VR bevorzugt werden, da hier CS seltener auftritt, das Unfallrisiko geringer ist und die nonverbale Kommunikation leichter fällt [54]. Studien zeigen, dass intuitive Formate besser akzeptiert werden [55, 56]. Eine Ausnahme bilden Vergleiche zwischen AR-HMDs und AR-Tablet-Anwendungen, wobei AR-HMDs oft als vorteilhaft angesehen werden, da sie eine immersive 3‑D-Auseinandersetzung mit dem Modell ermöglichen [42, 44, 45].

## Ausblick

Die meisten Studien zur digitalen anatomischen Lehre sind noch sehr anatomiezentrisch. Die Integration klinischer Inhalte ist eher selten. Am häufigsten wurden anatomisch-radiologische Lehrinhalte umgesetzt [34–37, 47], teils auch in Verbindung mit Fallstudien [44]. Die qualitative Bewertung durch die Studierenden ist positiv. So konnte gezeigt werden, dass Bilder und Fallstudien realer Patient:innen die Sinnhaftigkeit der Lehreinheit für die Studierenden erhöht [37]. Im klinischen Umfeld werden MR-Anwendungen bereits für die Vorbereitung und Visualisierung chirurgischer Eingriffe und als Fernberatungsdienst in der Telemedizin [57–62] eingesetzt. Es fehlen noch Anatomie-MR-Lernansätze, die auf die Bedürfnisse eines modernen Studiums zugeschnitten sind und die Anatomie mit klinisch relevanten Konzepten verzahnen und dem Fortschritt in der Digitalisierung des Klinikalltags Rechnung tragen. Die Entwicklung solcher Methoden ist komplex, da Anatom:innen, Kliniker:innen und Informatiker:innen gemeinsam an der Umsetzung arbeiten müssen. Was oft von Anatom:innen und Kliniker:innen gewünscht wird, ist Haptik und die Möglichkeit, z. B. Gelenke von 3‑D-Modellen auch beugen zu können. Allerdings wird dann die Umsetzung von IT-Seite noch zeitaufwendiger und die Rechenkapazität mancher HMDs erschöpft. Damit finden sich gute Argumente für Pro- und Dissektion im Anatomieunterricht, da hier die Haptik ein wichtiger Teil der Lernerfahrung ist. In allen Studien, in denen die Dissektion berücksichtigt wurde, sprechen die Ergebnisse für deren Beibehaltung in der anatomischen Lehre. In manchen Studien konnten digital Strukturen visualisiert werden, die bei Körperspendern nur schwer zu erkennen sind, wodurch die Qualität der Präparation in Präparierkursen verbessert wurde [39, 44]. Digitale Tools sind hier wertvolle Zusatzmethoden die begleitend, vorbereitend und vertiefend eingesetzt werden.

Zusammenfassend lässt sich sagen, dass ein guter anatomischer Unterricht seinen historischen Wurzeln treu bleibt und gleichzeitig moderne Technologien und klinische Konzepte integriert.

## Fazit für die Praxis

Digitale Lehrmethoden …

- können die klassischen Lehrmethoden ergänzen, aber nicht ersetzen,
- sollten in longitudinalen Lernspiralen v. a. später im Kurrikulum eingesetzt werden,
- mit Head-Mounted Displays (HDMs) sollten nicht zu lang gestaltet sein,
- müssen intuitiv und einfach in der Handhabung sein,
- sollten die Anatomie mit klinischen Inhalten verknüpfen.
